# Pulmonary rehabilitation after exacerbation of bronchiectasis: a pilot randomized controlled trial

**DOI:** 10.1186/s12890-019-0856-0

**Published:** 2019-05-06

**Authors:** James D. Chalmers, Megan L. Crichton, Gill Brady, Simon Finch, Mike Lonergan, Thomas C. Fardon

**Affiliations:** 1 Scottish Centre for Respiratory Research, University of Dundee, Ninewells Hospital and Medical School, Dundee, Scotland; 2 Division of Molecular and Clinical Medicine, University of Dundee, Dundee, DD1 9SY Scotland

## Abstract

**Background:**

Pulmonary rehabilitation improves exercise capacity and reduces risk of future exacerbation in COPD when performed after an exacerbation. There have been no previous studies of post-exacerbation rehabilitation in bronchiectasis.

**Methods:**

Parallel group randomized controlled trial compared pulmonary rehabilitation (PR) to standard care (SC) in patients followed an antibiotic treated exacerbation of bronchiectasis. Patients were randomized following a 14 day course of antibiotics was completed. The primary outcome was 6-min walk distance (6 MW) at 8 weeks. Secondary outcomes were time to the next exacerbation, St.Georges Respiratory Questionnaire, COPD CAT score, Leicester cough questionnaire (LCQ) and FEV1 at 8 and 12 weeks post exacerbation.

**Results:**

Forty eight patients were enrolled but only 27 had exacerbations within 12 months of enrolment. Nine patients received pulmonary rehabilitation and 18 received standard care. The 6 MW improved significantly from post-exacerbation to 8 weeks in both groups, with no significant difference between PR and SC- mean difference of 11 m (95% CI -34.3 to 56.3,*p* = 0.6). Time to the next exacerbation was not significantly different hazard ratio 0.83 (0.31–2.19, *p* = 0.7). No significant differences were seen between groups in terms of LCQ, CAT, FEV1 or SGRQ between groups. An analysis of probability based on the patients enrolled suggested > 1000 subjects are likely be required to have an > 80% probability of observing a statistically significant difference between PR and SC and any such differences would be likely to be too small to be clinically relevant.

**Conclusions:**

This pilot study identified no significant benefits associated with pulmonary rehabilitation after exacerbations of bronchiectasis.

**Trial registration:**

NCT02179983, registered on Clinicaltrials.gov 29th June 2014.

## Background

Exacerbations have a major impact on prognosis in patients with bronchiectasis [1, 2]. Patients with frequent exacerbations experience a high risk of hospitalization, have significantly worse quality of life and an increase in mortality of 86% for patients experiencing 3 or more exacerbations per year [2, 3].

Exacerbations are inflammatory events that also increase the risk of cardiovascular complications [4, 5]. Exacerbation symptoms last for a median of 16 days and up to 1 in 5 patients have not recovered symptomatically 35 days after an exacerbation [6]. Forced expiratory volume in 1 s and peak expiratory flow rate fall at exacerbation and improve at a variable rate during recovery leading to reduced physical activity and impairment of quality of life [6, 7]. Physical activity and exercise capacity are key determinants of quality of life in bronchiectasis and have been most frequently evaluated using the 6 min walk test [8, 9].

Disease progression in bronchiectasis is associated with a reduction in exercise capacity. 6-min walk distance is correlated with lung function and extent of bronchiectasis on CT scan and was found by McDonnell et al. to reflect disease severity measured by the bronchiectasis severity index [7]. Patients with severe disease walked an average of 83 m less than patients with moderate disease and 198 m less than those with mild disease [7].

Therefore maximising exercise capacity following exacerbations of bronchiectasis can be seen as preventing an important element of the disease progression associated with exacerbations [10–12].

In COPD, it is accepted that performing pulmonary rehabilitation after exacerbations are acceptable and highly beneficial [13]. In the systematic review by Puhan et al., pulmonary rehabilitation reduced future hospital admissions (OR 0.22 95% CI 0.08–0.58, number needed to treat = 4) and reduced mortality (OR 0.28 95% CI 0.10–0.84, number needed to treat = 6) with substantial impacts on quality of life and a mean 6-min walk distance improvement of 77.7 [13, 14]. For this reason the use of pulmonary rehabilitation after exacerbations of COPD is accepted into clinical practice.

Pulmonary rehabilitation has shown benefits in bronchiectasis [15]. A recent systematic review by Lee et al. found 4 trials involing 164 participants which showed an improvement in incremental shuttle walk distance in trials in stable patients with improvement in health related quality of life immediately post intervention which faded by 6 months [16]. One study of 20 patients which attempted rehabilitation during hospital admission was identified but no studies were identified testing rehabilitation post-exacerbation [17].

The Tayside Rehabilitation in Bronchiectasis Exacerbations (TRIBE) study was designed as a pilot study to determine potential benefits of rehabilitation following exacerbations of bronchiectasis.

## Methods

The TRIBE study was a parallel group randomized controlled pilot trial. The study was prospectively registered at Clinicaltrials.gov- NCT02179983 and is reported according to CONSORT guidelines. The objective of the study was to determine the effectiveness of pulmonary rehabilitation in improving exercise capacity and health related quality of life following an exacerbation of bronchiectasis. As a pilot study, it was also intended to provide information to power a definitive future trial. The study was approved by the East of Scotland ethics committee and all patients gave written informed consent (13/ES/0062).

### Inclusion and exclusion criteria and patient enrolment

Patients were enrolled when clinical stable and gave written informed consent. At enrolment patients agreed to contact the investigators at the onset of their next exacerbation.

The inclusion criteria were bronchiectasis confirmed on High Resolution CT scan; clinically significant bronchiectasis confirmed by a respiratory physician and at least one documented exacerbation within the last year. Patients were required to be independently mobile and therefore able to undertake pulmonary rehabilitation if randomized to this arm.

The exclusion criteria were Inability to give informed consent to participate; age < 18 years; a primary diagnosis of Chronic Obstructive Pulmonary Disease; significant comorbidity that would limit the ability to undertake pulmonary rehabilitation - i.e. Cerebrovascular, cardiovascular or musculoskeletal disease; known cystic Fibrosis; aortic aneurysm; recent myocardial infarction (within previous year) or unstable angina and patients having undergone pulmonary rehabilitation in the previous year.

### Exacerbations

Exacerbations were defined as a sustained worsening of respiratory symptoms for > 48 h and a decision by a physician that antibiotic therapy is required [18]. EXACT-PRO diaries were used to validate the worsening of respiratory symptoms, but not as the definition of exacerbation for inclusion.

After onset of exacerbation patients contacted the investigators who provided a standardised antibiotic regimen for 14 days based on prior microbiology results according to international guideline recommendations [15]. Patients were then reviewed at day 14 (completion of antibiotic treatment) and were randomized at day 14 to either intervention or control.

### Randomization

Patients were randomized using sealed opaque envelopes at 1:1 ratio to either pulmonary rehabilitation or standard care. Standard care consisted of guideline concordant ongoing management including instruction in daily chest physiotherapy [19, 20].

### Pulmonary rehabilitation

Patients randomized to pulmonary rehabilitation received a structured rehabilitation programme at Kings Cross Hospital in Dundee. Each patient was prescribed an individual programme and joined a group of approximately ten participants.

There were two supervised sessions per week and two ‘homework’ sessions for a total of 6 weeks. The class started with a gentle warm up and then moved into their own individual exercise programme based on their assessment and personal goals. Each programme contained a mixture of cardiovascular training – e.g. treadmill, bike, walking and strength training with free weights. Patients were exercised at 80% VO2max for cardiovascular work and one repetition maximum (RM) aiming for 8–10 reps for strength. All exercises were supervised and progressed by specialist physiotherapists and respiratory nurses. The exercise sessions were followed immediately by a cool-down, again led by the physio.

All patients attending the class were also given the following group educational activities; Benefits of exercise; Relaxed abdominal breathing and controlled breathing; Chest clearance techniques; Pathology of chronic lung disease (including bronchiectasis); Practical demo/advice on inhalers; Medications; Self-management; Smoking cessation; Pacing and energy conservation; Nutrition; Welfare rights and benefits; Maintenance options.

All patients received individual instruction in breathing strategies and chest clearance techniques to aid self-management. At the end of the six-week block of pulmonary rehab, patients were all encouraged to attend local maintenance groups including Active for Life (in local leisure centres) or circuit classes in various community settings (all supervised).

### Endpoints

Endpoints were evaluated at baseline, onset of exacerbation, end of exacerbation (day 14), 8 weeks post-exacerbation and 12 weeks post-exacerbation.

The primary outcome was change in 6 min walk test distance from end of exacerbation (day 14 post antibiotic) to 8 weeks post-exacerbation.

Six min walk distance at 12 weeks post-exacerbation was a secondary endpoint. Additional secondary outcomes were; time to the next exacerbation, quality of life using the St Georges Respiratory Questionniare at 8 and 12 weeks, spirometry at 8 and 12 weeks, Cough symptoms measured using the Leicester cough questionnaire and COPD CAT questionnaire at 8 and 12 weeks and sputum microbiology.

As this was a pilot study, an additional objective was to identify the number of patients that would have to be recruited to demonstrate a clinically meaningful improvement in 6-min walk distance.

### Statistical analysis

Statistical analysis was performed using the Graphpad Prism v6 and R. Categorical variables are presented by frequencies and percentages and statistical differences were analysed using χ^2^ test or Fisher exact test when required. Continuous variables are presented as mean and standard deviation (SD) or median and interquartile range (IQR) when data are not distributed normally. Mean differences at each time point were compared using t-tests. As the objective of the study was to power a definitive future trial, no a priori power calculation was conducted. To produce estimates of how many patients would have to be recruited to achieve statistically significant results a semi-Bayesian approach, using frequentist tests but interpreting their results as probability distributions for parameter values, was used. These allowed for the uncertainty in the means and standard deviations estimated from the pilot data. Non-parametric bootstrap resamples of the data were drawn and used to construct empirical joint distributions of means and standard deviations. Simulated datasets of various sizes were then created from these distributions and used to estimate the probability of reporting statistically significant differences between group means for the 6-min walk test that were > 0 m, > 25 m, > 54 m, for different sample sizes. 25 m and 54 m were chosen as they are previously described minimum clinically important differences for the 6-min walk test [21, 22]. All results are presented for the intention to treat population with a sensitivity analysis shown excluding patients with deviations from the protocol (per-protocol analysis). We defined statistical significance as a two-tailed *p* < 0.05 for all analyses.

## Results

**Fig. 1:**
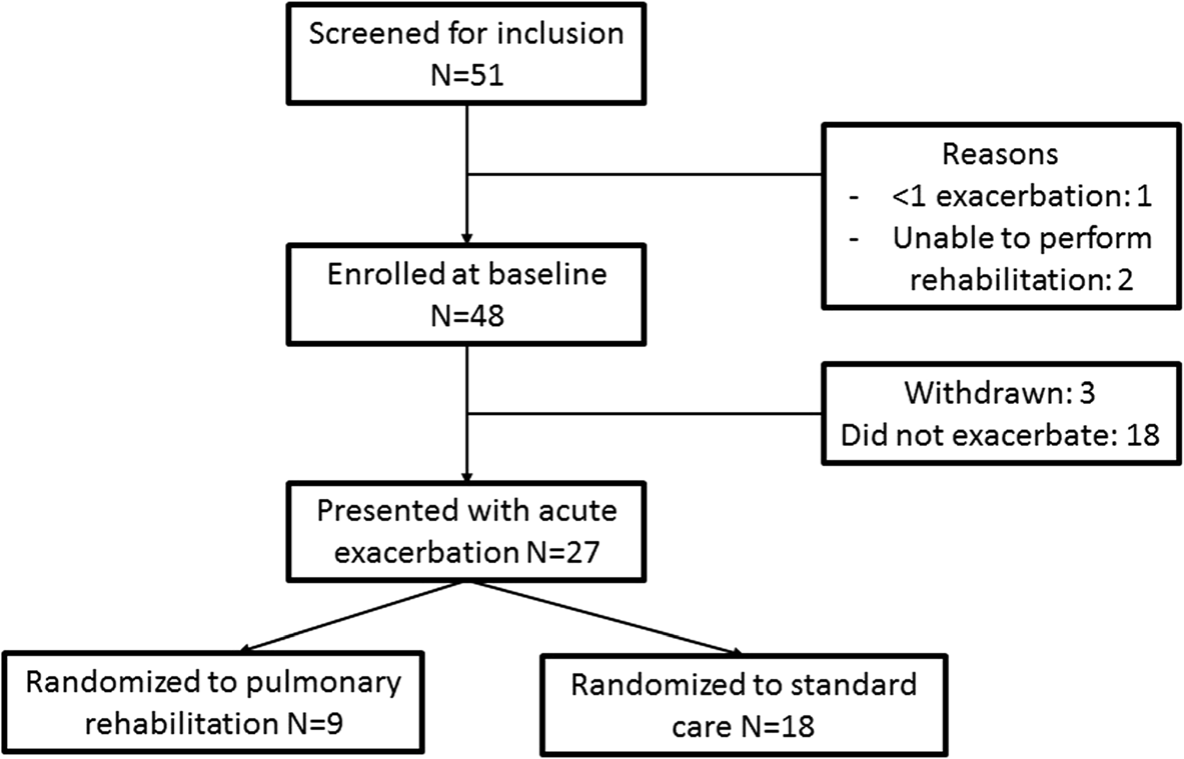
Flow chart of the study

Fifty one patients were screened and 48 patients were enrolled at baseline. 62.7% of patient were female and the median age was 68 years. Twenty seven patients attended with acute exacerbations and were randomized. Therefore 56% of patients enrolled could be randomized. Of those patients not attending within 12 months with exacerbations, 2 withdrew from the study prior to exacerbation while one patient was withdrawn by the investigators for failing to report exacerbations. The other patients did not have exacerbations during the observation period. The flow of patients through the study are shown in Fig. 1.

The characteristics of patients enrolled and the patients randomized in the study are shown in Table 1.

**Table 1:** Baseline characteristics of the study population

Characteristics	Patients enrolled at baseline	Patients randomized	Pulmonary rehabilitation (PR)	Standard care (SC)	*p*-value (comparing PR vs SC)
N	48	27	9	18	
Age (years)	68 (63–72)	68 (63–72)	68 (63–71)	68 (63–73)	0.8
Sex (% female)	31 (64.6%)	20 (74.1%)	5 (55.6%)	15 (83.3%)	0.2
Comorbidities					
Angina	4 (8.3%)	1 (3.7%)	0 (0%)	1 (8.3%)	1.0
Myocardial infarction	3 (6.3%)	0 (0%)	0 (0%)	0 (0%)	1.0
Osteoporosis	5 (10.4%)	4 (14.8%)	2 (22.2%)	2 (11.1%)	0.6
Anxiety	7 (14.6%)	5 (18.5%)	2 (22.2%)	3 (16.7%)	1.0
Diabetes	5 (10.4%)	3 (11.1%)	1 (11.1%)	2 (11.1%)	1.0
Hypertension	12 (25.0%)	9 (33.3%)	2 (22.2%)	7 (38.9%)	0.7
Smoking status					
Never	31 (64.6%)	18 (66.7%)	7 (77.8%)	11 (61.1%)	0.6
Ex	14 (29.2%)	8 (29.6%)	1 (11.1%)	7 (38.9%)	
Current	3 (6.3%)	1 (3.7%)	1 (11.1%)	0 (0%)	
Medications					
Inhaled corticosteroids	22 (45.8%)	16 (59.3%)	6 (66.7%)	10 (55.6%)	0.7
Macrolide	28 (58.3%)	17 (63.0%)	6 (66.7%)	11 (61.1%)	1.0
Other long term antibiotic	2 (4.2%)	1 (3.7%)	0 (0%)	1 (5.6%)	1.0
Inhaled antibiotic	1 (2.1%)	1 (3.7%)	0 (0%)	1 (5.6%)	1.0
Disease severity					
Exacerbations per year					
1	14 (29.2%)	4 (14.8%)	2 (22.2%)	2 (11.1%)	0.6
2	8 (16.7%)	8 (29.6%)	4 (44.4%)	4 (22.2%)	
3 or more	26 (54.2%)	15 (55.6%)	3 (33.3%)	12 (66.7%)	
Body mass index	27.3 (24.1–30.8)	27.7 (24.5–31.5)	30.7 (25.3–33.3)	26.8 (23.1–30.5)	0.3
FEV1	1.79 (1.19–2.11)	1.78 (1.19–2.06)	1.98 (1.10–2.50)	1.71 (1.25–2.05)	0.5
FEV1% predicted	81.5 (53.3–102)	81 (52–96)	76.0 (46.5–109)	83 (55.8–90.8)	0.4
FVC	2.76 (2.25–3.74)	2.69 (2.24–3.34)	2.92 (2.17–3.86)	2.67 (2.18–3.22)	0.5
6-min walk distance (m)	432 (334–497)	434 (348–500)	414 (280–494)	448 (400–507)	0.6
Bronchiectasis severity index					
Mild	9 (18.8%)	6 (22.2%)	2 (22.2%)	4 (22.2%)	0.9
Moderate	20 (41.7%)	10 (37.0%)	4 (44.4%)	6 (33.3%)	
Severe	19 (39.6%)	11 (40.7%)	3 (33.3%)	8 (44.4%)	
Microbiology at baseline					
*Haemophilus influenzae*	17 (35.4%)	9 (33.3%)	3 (33.3%)	6 (33.3%)	1.0
*Moraxella catarrhalis*	8 (16.7%)	5 (18.5%)	1 (11.1%)	4 (22.2%)	0.6
Enterobacteriaceae	7 (14.6%)	5 (18.5%)	2 (22.2%)	3 (16.7%)	1.0
*Pseudomonas aeruginosa*	6 (12.5%)	4 (14.8%)	2 (22.2%)	2 (11.1%)	0.6
Others	10 (20.8%)	4 (14.8%)	1 (11.1%)	3 (16.7%)	1.0

### 6-min walk distance

**Fig. 2:**
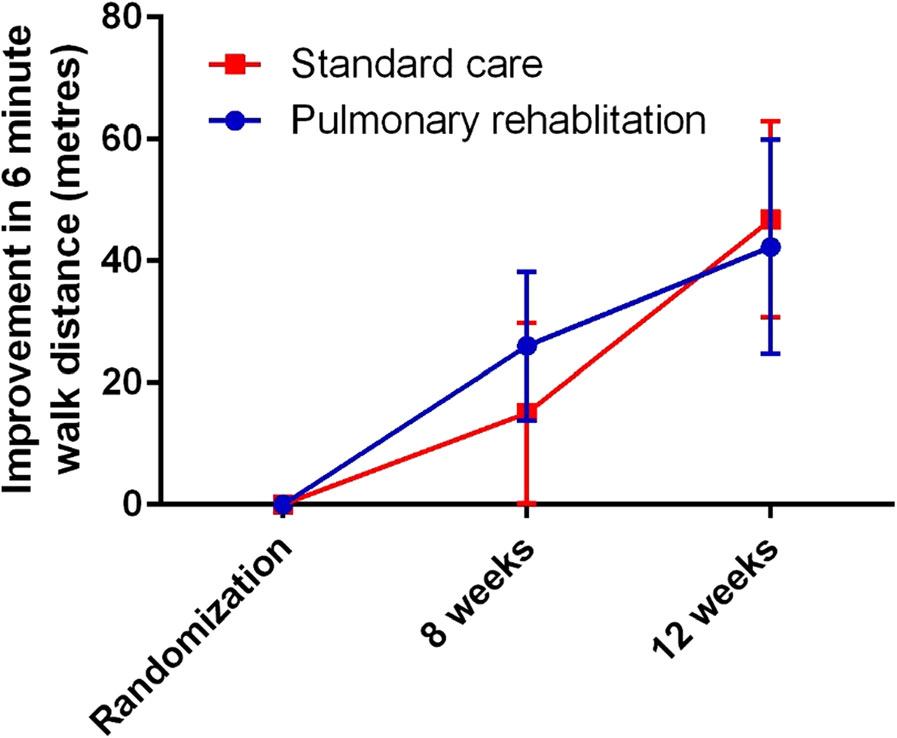
Improvement in 6-min walk distance from randomization at the end of antibiotic therapy to 8 weeks (primary outcome) and 12 weeks (secondary outcome). Data are presented as mean change from baseline with standard error

The 6-min walk distance improved from post-exacerbation to 8 weeks in both groups, with no significant difference between pulmonary rehabilitation and standard care. 6 min walk distance improved by 26 m (− 2 to 54.01) in the pulmonary rehabilitation group and 15 m (− 16.6 to 46.7) in the standard care group. The mean difference of 11 m (− 34.3 to 56.3), *p* = 0.6 was not statistically significant (Fig. 2).

There was similarly no statistically significant difference in 6 min walk distance at 12 weeks and in fact the improvement in walking distance was numerically greater in the standard care group compared to pulmonary rehabilitation (mean difference 4.6 m 95% CI − 59.9 to 50.7, *p* = 0.9).

### Secondary endpoints

**Fig. 3:**
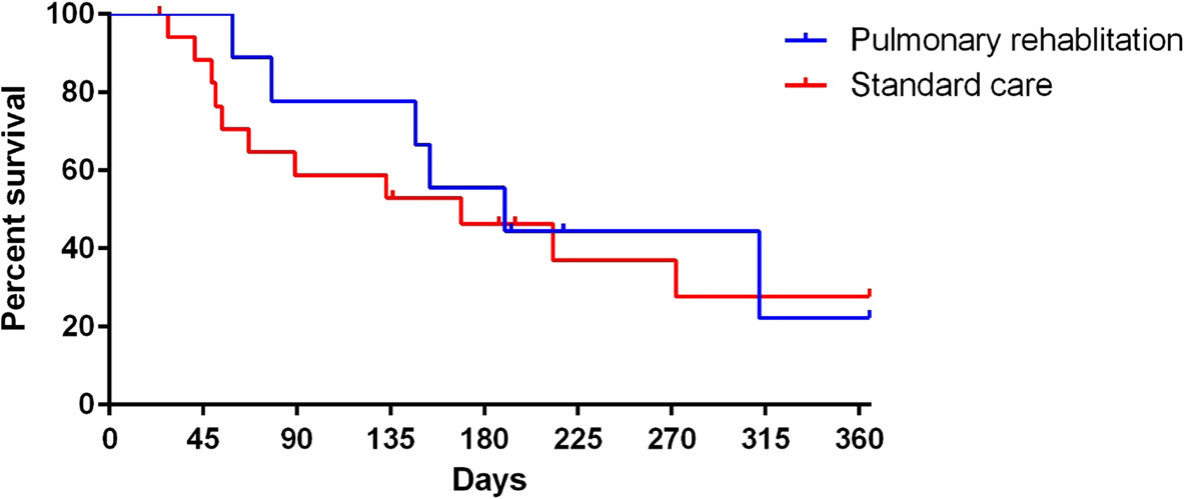
Kaplain-Meier survival curve showing time to the next exacerbation after randomization

Time to the next exacerbation was not significantly different between the two groups. The median time to next exacerbation was 169 days in the standard care group and 190 days in the pulmonary rehabilitation group. The hazard ratio was 0.83 (95% CI 0.31–2.19, *p* = 0.7) Fig. 3.

Cough symptoms improved by a mean of 0.83 points (+/− 1.24) in the pulmonary rehabilitation group compared to 0.91 points (+/− 0.61) in the standard care group at 8 weeks. The corresponding results at 12 weeks were 0.82 (+/− 1.1) and 0.51 (+/− 0.51), *p* = 0.8.

Symptoms measured using the CAT score improved by 2.1 points (+/− 1.6) in the pulmonary rehabilitation group and by 0.86 points (+/− 1.9), *p* = 0.6 at 8 weeks. Difference in symptoms was even greater at 12 weeks with an improvement of 2.89 points (+/− 1.9) in the pulmonary rehabilitation group and a worsening of symptoms by 0.6 points (+/− 2.5) in the standard care group. The mean difference of 3.5 points at this time point substantially exceeded the MCID for this scale but was not statistically significant (=0.3).

**Fig. 4:**
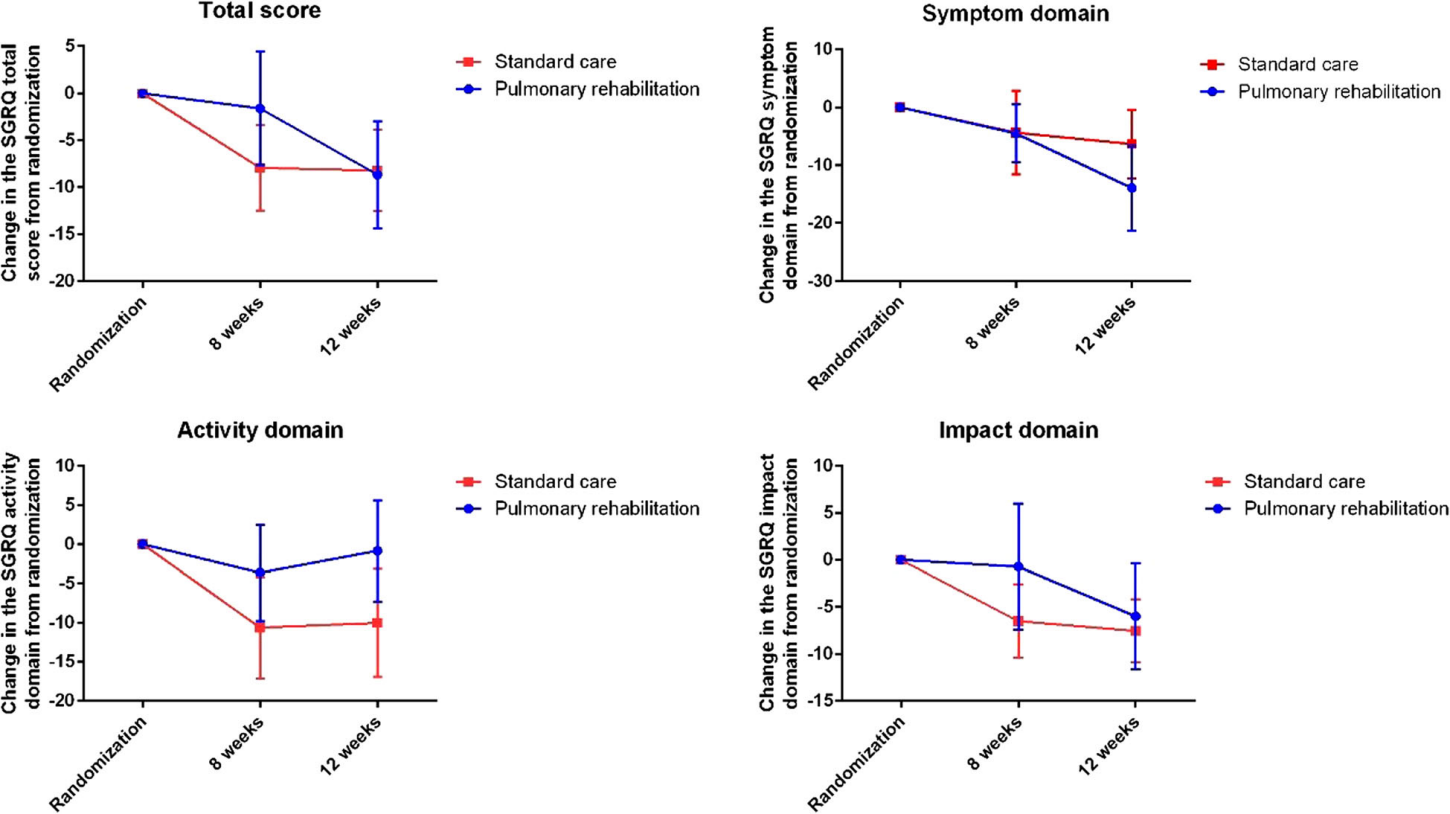
Changes from randomization in the St Georges Respiratory Questionnaire (SGRQ) across the 3 domains and the total score. No differences were statistically significant between the pulmonary rehabilitation and standard care groups at any time point. Data are presented as mean change with standard error

No changes in the St. Georges Respiratory questionnaire were statistically significant across the total score or the activity, impact or symptom domains (Fig. 4).

FEV1 improved by 100 ml (+/− 56 ml) in the pulmonary rehabilitation group compared to 21 ml (+/− 33 ml) in the standard care group at 8 weeks,*p* = 0.2 with no difference evidence at 12 weeks (*p* = 0.4).

Sputum microbiology showed no differences between groups.

The per-protocol analysis for the primary endpoint found a mean difference of 24 m (− 28.9 to 76.9 m), *p* = 0.4. No significant differences were observed in any other endpoints in the per protocol analysis. No safety issues were identified in the study.

### Power calculation

The data collected suggested that large differences between groups exceeding the reported MCIDs of 25 or 54 m in the 6- min walk test were unlikely. The probability of rehab leading to improved outcomes (based on the estimated mean difference and using a common standard deviation to calculate the standard error on the difference between the group means) is 0.77.

This translates into a 17% probability that rehabilitation improves mean 6 min walk test by more than 25 m, and a 0.2% chance that the difference is greater than 54 m.

**Fig. 5:**
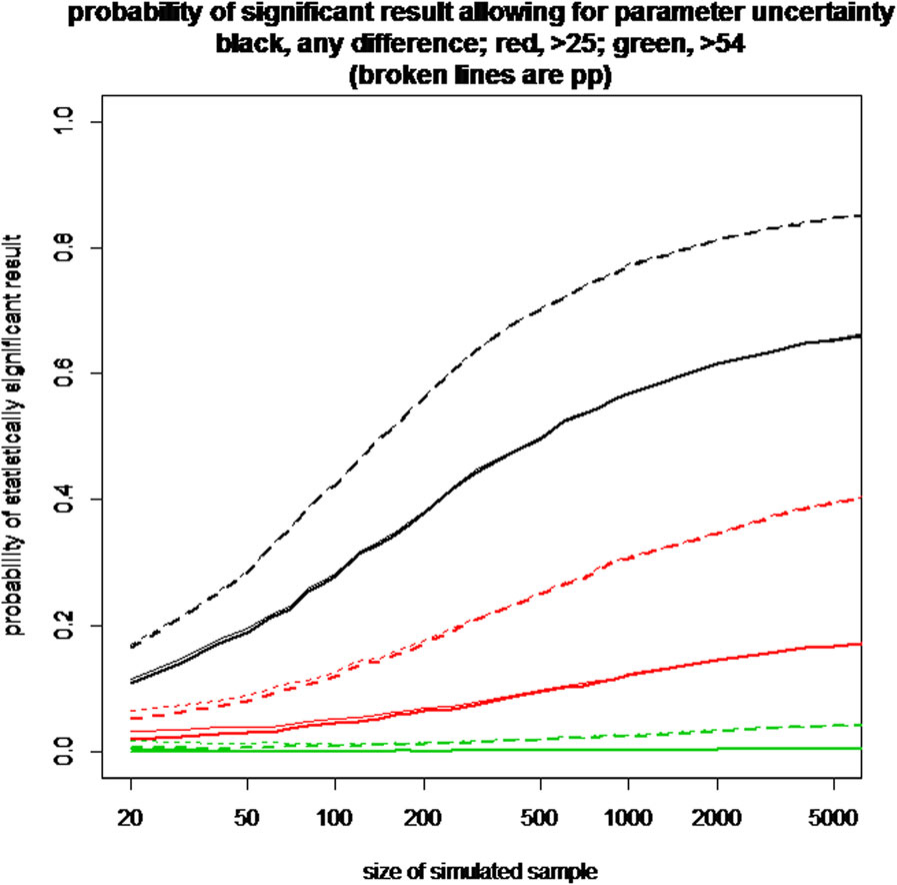
Probability of a significant difference comparing Pulmonary rehabilitation with standard care. The Y-axis shows the probability of a positive result and x-axis shows the required sample size. The colour of lines are as follows BLACK: any difference between groups is considered meaningful RED = a difference of > 25 m is required, GREEN = a difference of > 54 m is required. The broken lines indicate per-protocol analysis with solid lines indicating the intention to treat population

A full assessment of the probability of the study producing a statistically significant result for a clinically meaningful effect size needs to allow for uncertainty in both the estimate of the difference between the mean outcomes for the two treatments and the estimated common standard deviation around those means. It also needs to respect the correlation between those estimates. Uncertainty in the proportion completing per protocol has a smaller effect. The figure below shows that this probability continues to climb as the sample size increases past 2000, but the only scenario where the probability reaches 80% occurs when only the per protocol data is used and any significant difference, no matter how small, is considered a success. Even under those conditions, more than 1000 participants would need to be recruited (Fig. 5).

## Discussion

Pulmonary rehabilitation is an established intervention for the management of multiple respiratory disorders including COPD, interstitial lung disease and bronchiectasis [15, 23, 24]. For stable patients with bronchiectasis pulmonary rehabilitation has been strongly recommended by the 2017 European Respiratory Society Guidelines [15]. This was on the basis of a meta-analysis of 4 trials which each showed consistent benefits in terms of improved exercise capacity, improved symptoms and in one study, a reduction in exacerbations [16].

Our study was designed to evaluate whether the benefits of pulmonary rehabilitation would extend to patients following an exacerbation of bronchiectasis. This was based, in part, on the observation that pulmonary rehabilitation after exacerbations of COPD carries substantial benefits including reduced risk of hospital admissions and mortality [14].

Our study did not demonstrate significant benefits associated with pulmonary rehabilitation. 6-min walking distance and symptoms improved significantly between the immediate post-exacerbation period and 8 and 12 weeks post-exacerbation, but there were no significant differences between groups. For the primary outcome of 6-min walk distance in particular, the difference of 11 m at 8 weeks is unlikely to be clinically meaningful and there was no benefit of rehabilitation at 12 weeks with numerically greater improvement in the standard care group.

Our study was designed as a pilot trial to power a future larger trial if we found evidence to support the hypothesis that post-exacerbation rehabilitation was beneficial. Our analysis of the 27 subjects enrolled determined that while there is inevitably some uncertainty about the possible treatment effect given the small number of subjects enrolled, the variation in rates in recovery of 6-min walk distance indicated that more than 1000 subjects would need to be enrolled to achieve a > 80% probability of showing a clinically meaningful effect.

Our data make it unlikely, therefore, that pulmonary rehabilitation will be beneficial in this population of patients with exacerbations treated with oral antibiotics. Our study did not enrol patients with severe exacerbations requiring hospitalization and so does not exclude the possibility of a treatment benefit in this, or other populations not included in the present study. Our study did suggest a clinically meaningful difference, albeit not statistically significant, in respiratory symptoms measured by CAT. With such a small sample size it should be acknowledged that these differences may be the result of chance but they suggest that symptomatic improvement should be investigated in future trials of recovery from exacerbations.

Ours is not the only study that has failed to show benefit of pulmonary rehabilitation post-exacerbation. Greening et al. studied a cohort of 389 hospitalized individuals with multiple respiratory diseases including 20 subjects with bronchiectasis [17]. They randomized patients to early hospital based rehabilitation or standard care in hospital (within 48 h of hospital admission) consisting of daily supervised strength and aerobic training and neuromuscular electrical stimulation. The early rehabilitation failed to produce any clinical benefits in this trial and mortality was increased in the group that received rehabilitation at 12 months. The mechanism for this is unknown but as discussed by Spruit et al., appeared not be directly related to the intervention [13, 17].

Important strengths of our study include the well characterised patient population, the exclusion of patients with co-existing COPD where the role of pulmonary rehabilitation is established and the use of multiple endpoints to evaluate efficacy. Our study has important limitations. The sample size is small as this was a pilot study. The number of subjects enrolled (48) should have been sufficient to randomize at least 20 subjects to each arm but despite enriching for a population of patients with at least 1 exacerbation in the previous 12 months only 27 (56%) patients had an exacerbation during the subsequent year. This experience is entirely consistent with recent clinical trials in bronchiectasis. The RESPIRE trials of inhaled dry powder ciprofloxacin, for example, enrolled patients with a history of 2 more exacerbations per year but despite this history of frequent exacerbations, as many as 67% of patients in RESPIRE 2 did not experience an exacerbation in the subsequent year [25–27]. The reasons for this remarkable discordance between exacerbation reporting prior to trials and during the course of trials needs to be investigated in future studies. Randomization results in a possibility of unequal distribution of patients between arms in small studies and we experience this problem in TRIBE. Indeed twice as many subjects were randomized to the standard care arm as the intervention arm. Nevertheless the power calculations indicate that potentially thousands of patients would have been required to show clinically meaningful differences in the study. Our study was designed prior to the widespread use of the quality of life bronchiectasis questionnaire and so the SGRQ and CAT score were used instead. We are aware that these were not originally designed for bronchiectasis and future studies should establish if symptoms and quality of life improve when using bronchiectasis specific tools [28–30]. Our study was performed prior to the new consensus definition of bronchiectasis exacerbations [18]. Although compliance of those patients randomized to pulmonary rehabilitation was excellent for the supervised visits, we did not monitor adherence to the home training sessions. Adherence to pulmonary rehabilitation is suboptimal in clinical practice and so the results of randomized trials do not always transfer into benefits in real life.

## Conclusion

Our pilot randomized trial identified no statistically significant improvement in exercise capacity, symptoms or quality of life with pulmonary rehabilitation after exacerbations. This pilot data suggests that any benefits of pulmonary rehabilitation in this setting are likely to be too small to be clinically meaningful.

## Abbreviations

6 MW: 6 min walking test
CAT: COPD assessment test
CI: Confidence intervals
COPD: Chronic obstructive pulmonary disease
CT: Computed tomograph
FEV1: Forced expiratory volume in 1 s
FVC: Forced vital capacity
IQR: Interquartile range
LCQ: Leicester cough questionnaire
PR: Pulmonary rehabilitation
SC: Standard care
SGRQ: St Georges Respiratory Questionnaire
